# Fractal Dimension Analysis of Transient Visual Evoked Potentials: Optimisation and Applications

**DOI:** 10.1371/journal.pone.0161565

**Published:** 2016-09-06

**Authors:** Mei Ying Boon, Bruce Ian Henry, Byoung Sun Chu, Nour Basahi, Catherine May Suttle, Chi Luu, Harry Leung, Stephen Hing

**Affiliations:** 1 School of Optometry and Vision Science, UNSW Australia, Sydney, New South Wales, Australia; 2 School of Mathematics and Statistics, UNSW Australia, Sydney, New South Wales, Australia; 3 Centre for Eye Research Australia, Royal Victorian Eye and Ear Hospital, East Melbourne, Victoria, Australia; 4 Department of Surgery (Ophthalmology), Melbourne Medical School, The University of Melbourne, Parkville, Victoria, Australia; 5 Park Road Eye, Hurstville, New South Wales, Australia; Universidad de Jaen, SPAIN

## Abstract

**Purpose:**

The visual evoked potential (VEP) provides a time series signal response to an external visual stimulus at the location of the visual cortex. The major VEP signal components, peak latency and amplitude, may be affected by disease processes. Additionally, the VEP contains fine detailed and non-periodic structure, of presently unclear relevance to normal function, which may be quantified using the fractal dimension. The purpose of this study is to provide a systematic investigation of the key parameters in the measurement of the fractal dimension of VEPs, to develop an optimal analysis protocol for application.

**Methods:**

VEP time series were mathematically transformed using delay time, *τ*, and embedding dimension, *m*, parameters. The fractal dimension of the transformed data was obtained from a scaling analysis based on straight line fits to the numbers of pairs of points with separation less than *r* versus log(*r*) in the transformed space. Optimal *τ*, *m*, and scaling analysis were obtained by comparing the consistency of results using different sampling frequencies. The optimised method was then piloted on samples of normal and abnormal VEPs.

**Results:**

Consistent fractal dimension estimates were obtained using *τ* = 4 ms, designating the fractal dimension = *D*_*2*_ of the time series based on embedding dimension *m* = 7 (for 3606 Hz and 5000 Hz), *m* = 6 (for 1803 Hz) and *m* = 5 (for 1000Hz), and estimating *D*_*2*_ for each embedding dimension as the steepest slope of the linear scaling region in the plot of log(C(*r*)) vs log(*r*) provided the scaling region occurred within the middle third of the plot. Piloting revealed that fractal dimensions were higher from the sampled abnormal than normal achromatic VEPs in adults (p = 0.02). Variances of fractal dimension were higher from the abnormal than normal chromatic VEPs in children (p = 0.01).

**Conclusions:**

A useful analysis protocol to assess the fractal dimension of transformed VEPs has been developed.

## Introduction

Visual evoked potentials (VEPs) are electrophysiological signals in response to temporally modulated stimuli recorded by electrodes located on scalp overlying the visual cortex. Such VEPs mainly reflect functional integrity of the visual pathway processing stimuli in the central visual field [1]. Visual evoked potentials may be used to indicate the health, normality and maturity of the visual system [1–3].

The International Society of Clinical Electrophysiology and Vision (ISCEV) has defined protocols for clinical assessment and the analysis of the VEP waveforms [1]. Transient VEPs record visual processing following a stimulus change. A typical VEP signal has a waveform appearance with peaks and troughs. The ISCEV protocol specifies the measurement of the amplitudes of the peaks and troughs and their latency, i.e., the time since the stimulus change. These measurements provide information about the speed and strength of processing arising from the cortical generators of the VEP components [1].

More generally, the VEPs contain fine detailed and possibly non-periodic structure. The importance of these aspects for normal visual processing is not yet clear. One method that has been used recently to provide an understanding of non-periodic structures in VEPs is the measurement of the fractal dimension of the time series [4–7]. Fractal dimensions may be used to quantify the complexity of a pattern by characterising how detail in the pattern changes with the scale in which it is measured. Fractal dimension measurements have been proven to be useful for quantifying chaotic, or non-linear deterministic, dynamical systems. Fractal structures may be identified in chaotic time series using Grassberger and Procaccia’s algorithm [8, 9]. The fractal dimension provides an indication of the minimum number of first order differential equations, equivalent to the minimum number of dynamical variables required to model the behaviour of the dynamical system [8, 9]. In previous studies the VEP has been found to behave as part of a nonlinear deterministic dynamical system characterized by fractal dimensions [4–7]. In the framework of nonlinear dynamical analysis, the VEP is regarded as a time series which images the electrophysiological activity of the visual system in the time domain for a fixed window of time. The VEP time series as specified in the ISCEV protocol is an ensemble average across multiple observations with the same stimulus. The averaging is necessary to remove EEG activity that is not related to the stimulus change.

When applied to electrophysiological signals such as the EEG or VEP, the fractal dimension quantifies the average relative complexity of patterns of communication between cortical neurons. This complexity, represented by the number of dynamical variables, may be related to the number of neuronal populations (source generators) [10, 11] or it may be related to structure in the electrophysiological activity of single neuronal populations [12]. It should be noted, however, that the transient VEP, recorded according to the ISCEV protocol is averaged to minimise the input of noise. Consequently, the complexity of all brain dynamics are not being investigated, but a subset of dynamics restricted to the visual system [13]. The fractal dimension measurement as applied in this study is not as a test for chaotic time series, but as a reproducible and reliable measurement for characterizing VEPs that is complementary to amplitude and latency measures.

Commercial VEP systems typically record transient VEPs using 1000 Hz sampling frequency. While sampling frequency does not impact greatly on the evaluation of amplitude and latency, the number of data points in the time series limits the accuracy of the estimate of the fractal dimension using Grassberger and Procaccia’s algorithm [14, 15]. A higher number of data points and higher sampling frequencies allow higher fractal dimensions to be estimated with improved accuracy [14, 15]. Abnormal visual systems might have higher dimensions than normal systems hence the use of sampling frequencies >1000 Hz might be useful. Higher sampling frequencies may also facilitate the examination of finer detail in the VEP through other measures such as detrended fluctuation analysis [16].

One potentially useful clinical application for the fractal dimension would be to assist in the differentiation of normal and abnormal visual systems. However, when computing the fractal dimension, there are a number of parameters and decision rules that must be optimised as the characteristics of the time series and the underlying dynamics of the system generating the time series on which these parameters depend are not known *a priori*. If the fractal dimension is to find application in the analysis of clinical signals, a consistent set of protocols for its measurement must be devised. In previous work, the values of these parameters were optimised for the analysis of transient VEPs recorded with a 1000 Hz sampling frequency from children and adults with normal VEPs [5, 6, 17] but not for other sampling frequencies or for VEPs recorded from people with abnormal visual systems. This provides the primary motivation in this paper for the systematic investigation of the parameters in the measurement of the fractal dimension of VEPs in an effort to develop an optimal analysis protocol for clinical application. Our criteria for an optimal analysis protocol are that it yields fractal dimension estimates that are comparable with previous work and produces *D*_*2*_ estimates that are similar across different sampling frequencies.

## Materials and Methods

This study focuses on developing protocols for one fractal dimension measurement, the correlation dimension (*D*_*2*_) which is estimated using Grassberger and Procaccia’s algorithm [8, 9], for VEPs recorded using different commercially available sampling frequencies. This study was conducted in two phases. In the first phase, in recognition of the sequential nature of the estimation of *D*_*2*_ using the Grassberger and Procaccia algorithm, we developed the analysis protocol in stages such that later steps in the algorithm were only investigated after earlier steps were optimised. In the second phase, we applied the optimised protocol to a sample of VEPs recorded from adults and children, to investigate its ability to differentiate between VEPs drawn from normal and abnormal visual systems. As such, in both phases 1 and 2, the fractal dimension, *D*_*2*_, was the primary outcome variable that was analysed statistically. However, the VEPs were described in terms of their standard ISCEV components: CI (first positive peak component), CII (first trough component) and CIII (second positive peak component) latencies and amplitudes. CI amplitude was calculated baseline-to-peak. CII amplitude was assessed as the peak-to-peak amplitude CI to CII. CIII amplitude was assessed as the peak-to-peak amplitude CII to CIII. Peak latency of each component was calculated as the time taken between stimulus onset and when the highest point in the peak, or lowest point in the trough. Components were repeatable if the peaks and troughs identified had latencies that were within 10% of the longest latency for two successive recordings of the VEP to the same visual stimulus [6].

In Phase 1, the key parameters investigated in the measurement of *D*_*2*_ are the delay time *τ* and the embedding dimension *m*. *D*_*2*_ is estimated from scaling behaviour at fixed values of *τ* and *m*. Custom software was written using Matlab (The Mathworks, Inc., Massachusetts, United States) to determine suitable values for these parameters. In Phase 2, the optimised method was then piloted on samples of normal and abnormal VEPs. The persons carrying out the analysis were unaware whether the VEPs they were analysing were drawn from normal or abnormal visual systems.

The study protocol, including the consent procedure, was approved by human research ethics committees (UNSW Australia Human Research Ethics Committee approval number: 09364 and SingHealth CIRB Secretariat Reference Approval Number: R1083/98/2013). All participants were provided with study participant information and consent forms and were given the opportunity to ask questions of the researchers to clarify any concerns. As the children had varying levels of reading and writing ability, the information in the consent form was conveyed to the children by the researchers either verbally or in written format. After agreeing to participate in the study, the participants >18 years of age gave their written consent and signed the consent forms. In the case of child participants, the guardian/parent of each child gave their written consent and signed the consent forms after agreeing to allow their child to participate in the study. The child participants provided their assent, verbally, to the best of their ability to understand. The original signed copies of the informed consent forms are stored by the investigators in a secure location in accordance with the human research ethics committee documentation. The tenets of the Declaration of Helsinki were followed throughout.

### Visual evoked potential sample characteristics

Samples of VEPs were drawn randomly from a dataset which included VEPs recorded from children and adults with normal and abnormal visual systems. Normal vision was defined as having normal visual acuity (6/6 Snellen visual acuity or better), no congenital colour vision deficiencies (Ishihara pseudoisochromatic colour vision screening test was used), normal stereopsis (<50 seconds of arc) and no sign of ocular abnormality (evaluated using direct ophthalmoscopy). The abnormal VEPs were recorded in children and adults with known abnormalities of visual pathway function including amblyopia, strabismus and central retinal artery occlusion [18]. The diagnosis of normality or otherwise was confirmed by ocular and visual system evaluation by registered optometrists and ophthalmologists.

The VEPs were recorded in response to stimuli that were either black-white or chromatic (magenta-cyan) gratings of low spatial frequency—1 cycle per degree (cpd) in the children and 2 cpd in the adults—on gamma corrected and calibrated cathode ray tube monitors. All visual stimuli were presented pattern onset-offset (on 100 ms and off 400 ms) at a 2 Hz temporal frequency. The electrode montage employed was according to 2004 ISCEV standards [19] so scalp electrodes were applied as follows; active electrode at Oz, reference electrode at Cz and the ground electrode at Fz. Two VEPs were recorded in response to each stimulus condition. Each VEP comprised one second sweep duration and the average of 30 sweeps. Each VEP was recorded on one of three commercial medical visual electrophysiology systems at their maximum sampling frequencies: Medelec Synergy (Radiometer Pacific, Sydney, Australia; 1000 Hz) and Espion (Diagnosys LLC, Lowell, Massachusetts; two systems, 3606 Hz and 5000 Hz).

### Application and optimisation of parameters for dimension estimation

The application of Grassberger and Procaccia’s algorithm to transient VEPs has been described previously and is briefly reviewed here [5, 6]. In this method, the VEP is regarded as a time series that is one observation of the dynamics of a deterministic system. For VEPs the deterministic system would be a mathematical model for the average electrophysiological processing of the visual system following a given stimulus change. If a system is deterministic, then any activity that occurs at one point in time must be dependent on activity that occurred earlier in the time series. The time evolution of the system that produces the VEP can be represented as a path, or phase space trajectory, in an abstract mathematical space called phase space. Using the time series for just one of the components (in this case the average observation that is the VEP) of the system, it is possible to reconstruct a path that shares the same invariant properties (such as dimension) as the full phase space trajectory. This process of reconstruction is called phase space embedding. If the reconstructed phase space has *m* dimensions, then each reconstructed phase space coordinate *X*_*i*_ is an *m* component vector which is obtained from the time series *y(t*_1_), *y(t*_2_),… by the prescription *X*_*i*_ = (*y(t*_i_), *y(t*_i_+τ), *y(t*_i_+2*τ*),…, *y(t*_i_+((*m*-1)*τ*) where *m* and *τ* are constant parameters, referred to as the embedding dimension, and the delay time, respectively. The index *i* denotes ordering in time. Phase space trajectories of deterministic dynamical systems usually evolve towards a particular set of coordinates called an attractor, although this may be a transient attractor in the case of VEPs, and the dimension of the attractor is less than that of the full phase space. Grassberger and Procaccia’s algorithm can be used to characterise the phase space filling properties of the path. The dimension is obtained by covering the set with boxes of a given size (*r*) and then computing the probability *p*_*i*_(*r*) (equivalent to the relative frequency in sufficiently large data sets) of having a point of the set in the *i*th box. The correlation dimension (*D*_*2*_) for a set of points is formally defined as:

$$D_{2}=\underset{r\to \infty }{\lim}\frac{\log\sum_{i}p_{i}^{2}(r)}{\log r}$$ (Eq 1)

where $p_{i}^{2}(r)$ is the probability of finding a pair of points in a box of size *r*. The limit *r* to zero is not amenable to real world data and instead some scaling relations are used. Grassberger and Procaccia [8, 9] found that for small values of *r* and for sufficiently large numbers of data points, *N*, the probability of having a pair of points in a box of size *r* is the same as the probability of having a pair of points with separation distance less than *r*. This latter probability is the correlation sum described by the following formula:

$$C(r)=\underset{N\to \infty }{\lim}\left(\frac{1}{\left(N(N-1\right)}\right).number\;of\;pairs\;of\;po\mathrm{int}s\;with\;separation<r$$ (Eq 2)

For small *r*, the correlation sum grows according to a scaling relation $C(r)∼r^{D_{2}}$. This scaling relation is only valid if *r* is small and *N* is large and this needs to be borne in mind in applications. Rearranging the scaling relation and taking logarithms of both sides, shows that *D*_*2*_ may be approximated by log (C(*r*))/log(*r*), which is usually approximated as the slope of the straight line scaling region in a plot of log(C(*r*)) vs log(*r*).

When used to estimate the correlation dimension of a time series, the time series is embedded into a range of phase space dimensions, *m*. The dimension *D*_*2*_ is estimated for each embedding dimension and plotted as a function of *m*. If the function plateaus, then the value at which it plateaus provides an estimate of the fractal dimension of the system, provided that the plateau occurs before the threshold value limited by the number of data points.

It can be seen that a number of key decisions must be made. As sampling frequency is varied, what are appropriate values for the delay time *τ*? From which range of embedding dimensions should the fractal dimension, *D*_*2*,_ be measured? How should the straight line scaling region be identified in the plot of log(C(*r*)) vs log(*r*)? These questions are considered below.

#### Considerations in the selection of delay time, *τ*

If *τ* is too small, then *y(t*_i_+*τ*) is almost the same as *y(t*_i_) and therefore a reconstructed attractor will lie nearly on a straight line with *D*_*2*_ = 1. If *τ* is too large, the time series points may be too widely separated in time to be considered as components of a single phase space vector. In this case there may be no discernible structure in the reconstructed phase space trajectory [20]. Therefore *τ* should be selected so that the reconstructed trajectory is not stretched out along the diagonal in the embedding space [21, 22]. For embedding dimensions less than four this can be seen in a simple visual plot of the trajectory [5]. In previous studies, for VEP data recorded at 1000 Hz sampling frequency, *τ* of 4 or 6, equivalent to 4 or 6 ms in the time domain, permitted the fractal dimension of VEPs in children and adults to be estimated and distinguished from each other [5, 6, 17]. In this study delay times of *τ* = 1, 3, 6, 9, 13, 16, 19, 22 and so on were trialled on VEPs recorded at sampling frequencies >1000 Hz. Our guiding principle was to select the smallest values for *τ* that revealed structures other than thin lines stretched out along the diagonal in the reconstructed phase space trajectories with very little change in structure for a range of increasing *τ* [21].

#### Considerations regarding the scaling analysis

**Fig 1:**
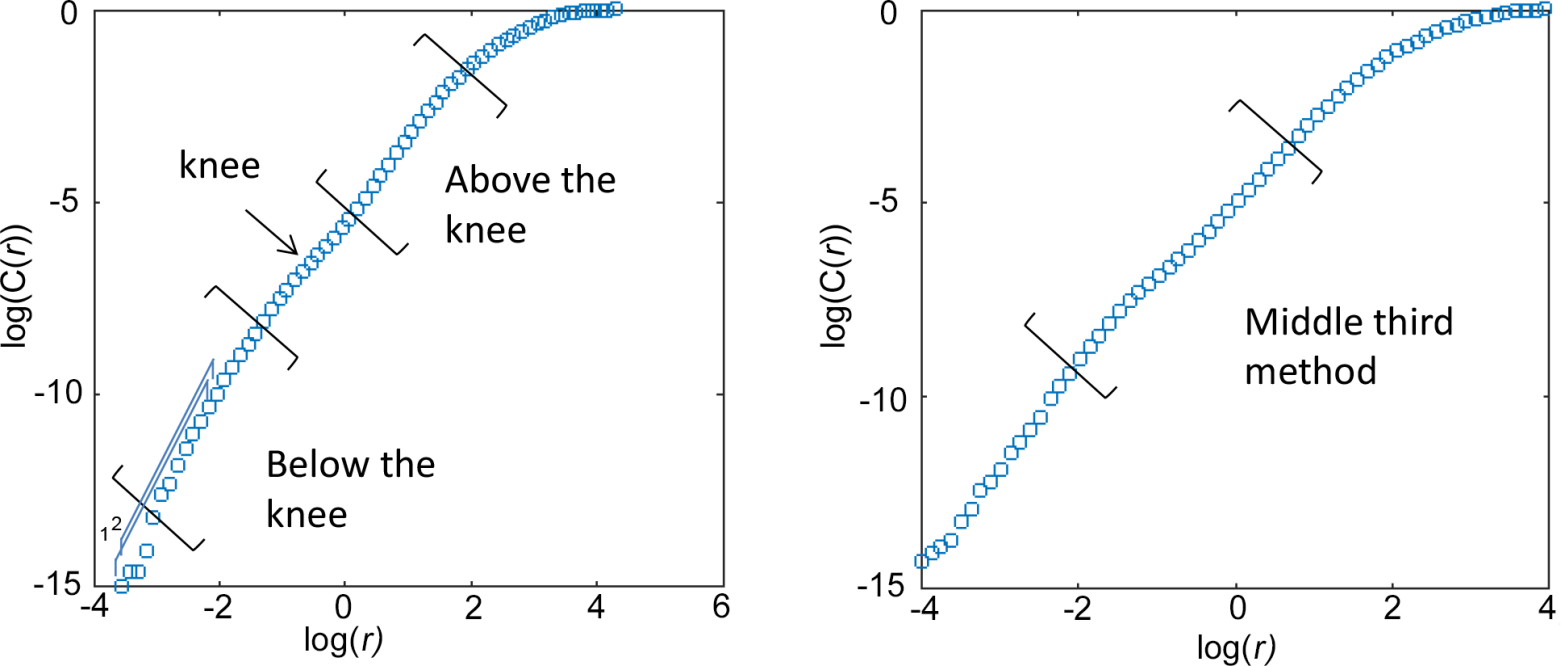
Two methods of finding the scaling region in sample plots of log(C(*r*)) vs log(*r*) are illustrated: Above and below the knee, and the Middle Third Rule. The numbers in blue, “1, 2”, indicate two consecutive running average slopes of 12 consecutive data points.

The scaling region within the plot of log(C(*r*)) vs log(*r*) does not always cover the entire plot. Recall that the scaling relation was dependent on *r* being small and *N* being large however there is a minimum *r*, below which no pairs of points would be in a box, and there is a maximum *r*, above which all pairs of points would be in the box. Slopes should be calculated from a range of box sizes, *r*, between these extremes. Henry et al. [21] suggested using the middle third of the data to estimate slope (see Fig 1). The criterion of the middle third rule objectively avoids the smallest scales for which fluctuations are great and the largest scales that are constrained by the finite number of data points in a discrete time series. This is an objective measure but it may cross changes in slope, such as for those plots of log(C(*r*)) vs log(*r*) which contain a “knee”, where the plot appears to bend in a manner similar to a knee. Tsonis et al. [23] showed in a plot of slope as a function of log(*r*) that very small scales tend to show large fluctuations in slope. Tsonis et al. [23] does not suggest using the middle third to estimate *D*_*2*_ but agreed that *D*_*2*_ should be estimated from an intermediate region, and suggested using the widest plateau in the plot of slope as a function of log(*r*) as it would be the most stable scaling region. Note that this will not typically occur for the smallest and largest values of *r*.

Some researchers have suggested measuring the slope above, below, or at a tangent along the knee [24]. The importance of the presence or absence of a “knee” in relation to VEP data is not known and is worth further investigation. One possible explanation for a knee is that the data is noise on scales *below* the knee but is deterministic on scales above the knee. In this case we would expect to measure *D*_*2*_ = *m* below the knee since noise is space filling, or approaching *m* in the case of data- limited noise, and *D*_*2*_<*m* above the knee as deterministic structure is expected to be non-space filling.

To estimate *D*_*2*_ for each embedding dimension, *m*, we first computed a running average slope over 12 consecutive data points in the plot of log(C(*r*)) and log(*r*), and then made plots of the running average slope as a function of log(*r)*. Three strategies were then considered: (i) If a knee was present in this plot, slope was estimated above the knee and below the knee separately (see Fig 1) providing two values for *D*_*2*_ for a given *m* based on the corresponding plateau values. (ii) *D*_*2*_ for a given *m* was estimated as the largest value within the widest plateau region. (iii) If the widest plateau in the plot of running average slope as a function of log(*r*) occurred within the middle third, *D*_*2*_ for a given *m* was estimated as the largest value from the plateau within the middle third.

To determine if the values of *D*_*2*_ obtained for each embedding dimension were indicative of either deterministic structure or noise, we plotted *D*_*2*_ as a function of the embedding dimension *m*. The characteristic feature of deterministic structure in this plot is that *D*_*2*_ limits to a ceiling value less than *m*. This shows up as a plateau in the plot of *D*_*2*_ versus *m*. To determine whether *D*_*2*_ reached a ceiling value in this plot we measured a plateau index PI [5] defined as:

$$\text{PI}=D_{2}\left(m*\right)-D_{2}(m*-1)$$ (Eq 3)

where m* is the maximum *m* allowed by the data according to the Eckmann-Ruelle criteria [14],

$$\text{m}*\leq \left(2{\text{log}}_{10}\left(N\right)\right)$$ (Eq 4)

where *N* is the number of data points We took PI<0.3 as the indicator that *D*_*2*_ had reached a ceiling value [5].

Our criterion for an optimal scaling method is that it results in an unambiguous measure of slope for all VEPs and yields estimates of *D*_*2*_ that are comparable with previous work [5, 6]

#### Comparability of fractal dimension estimates for different sampling frequencies, embedding dimension considerations

To check whether the parameters and rules yielded by the above strategies are unaffected by sampling frequency, it is necessary to compare *D*_*2*_ for VEPs identical in every way except for the sampling frequency using the optimised method. A higher sampling frequency provides more data points, *N*, and hence a greater maximum embedding dimension, m*, (see Eq 4), and a greater possible correlation dimension, *D*_*2*_ ≤ m*. The fractal dimension *D*_*2*_ was computed for VEP time series at their originally recorded sampling frequency, and half that (achieved by extracting every second point from the original time series): VEPs with 5000 Hz and 3606 Hz sampling frequencies were halved to 2500 Hz and 1803 Hz respectively. From the Eckmann-Ruelle criteria, Eq 4, we estimate upper bounds, m* = 7 for 3606 and 5000 Hz sampling frequencies, m* = 6 for 1803 and 2500 Hz sampling frequencies and m* = 5 for 1000 Hz sampling frequencies for our calculations.

### Statistical analysis

This study was carried out in two phases and the primary outcome measure of interest was *D*_*2*_ for each of the phases. The purpose of the first phase was to determine the protocol; family-wise Type 1 error was controlled by using a Bonferroni-Sidak correction to adjust the α by the total number of statistical analyses conducted, 3, to 0.017. In the second phase, in which the protocol was piloted on samples of VEPs drawn from normal and abnormal visual systems, an Analysis of Variance test with Bonferroni corrections of the post-hoc paired comparisons were conducted, to control family-wise Type 1 error.

## Results

### Phase 1: Optimising the analysis protocol

#### Delay time, *τ*

**Fig 2:**
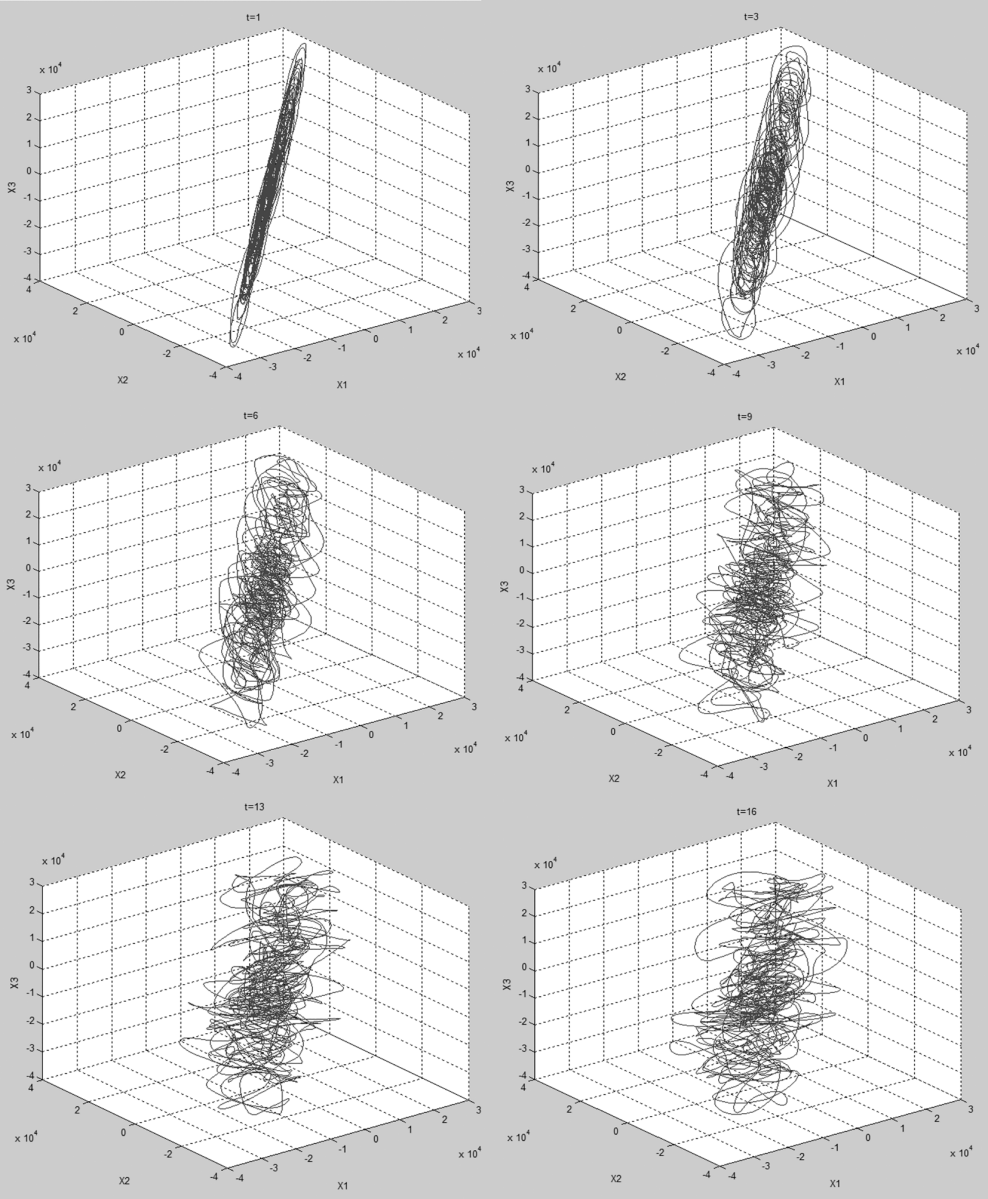
Reconstructed phase space trajectories of a VEP recorded with 3606 Hz sampling frequency embedded in 3 dimensional phase space using different values of *τ*. From top left to bottom right, *τ* = 1, 3, 6, 9, 13, 16, 19, 22.

Fig 2 shows typical plots of the reconstructed phase space trajectories for different *τ* values. The plots shown are from a VEP of a child with abnormal vision due to amblyopia (initials LS) recorded using a 3606 Hz sampling frequency in response to black and white sine wave gratings. For *τ* ≥ 16 data points (4.4 ms), the shape of the reconstructed trajectory appears to be topologically stable (i.e. has geometric properties preserved despite deformations such as stretching) under rotation when viewed in Matlab, and not simply stretched along the diagonal. Fig 2 and Table 1 show that while delay time in terms of number of data points changes with sampling frequency, the shortest useful delay time for VEPs recorded at 3606 and 5000 Hz sampling frequency is about 4 ms.

**Table 1:** Range of values for *τ* for VEPs recorded at >1000 Hz such that reconstructed phase space trajectories were not stretched along the diagonal such that fluctuations in the trajectory were evident.

Participant initials	Normal (N) or abnormal (AB) visual system	Sampling frequency (Hz)	Values of *τ* for which the structure of the phase space trajectory was visible in 3 dimensional phase space, and for which structure appeared morphologically similar	*τ* converted to ms (delay time / sampling frequency)
RI	AB	3606	16–24	4.4–6.6
AA	AB	3606	16–24	4.4–6.6
ET	AB	3606	16–24	4.4–6.6
EP	AB	3606	16–24	4.4–6.6
EP	AB	3606	16–24	4.4–6.6
LS	AB	3606	15–24	4.4–6.6
KL	N	3606	16–24	4.4–6.7
MG	N	3606	16–24	4.4–6.7
SH	N	3606	16–24	4.4–6.7
TZ	N	3606	16–24	4.4–4.8
DL	N	3606	15–20	4.2–5.5
S1	AB	5000	22–40	4.4–8 .0
S1	AB	5000	20–30	4.0–6.0

Shaded cells indicate abnormal VEPs.

#### The scaling analysis

In the log-log plots of C(*r*) versus *r*, a knee was present in 42 out of 47 cases. Two slopes were estimated, one below the knee and the other above the knee. The proportion of slopes with PI<0.3 (see Eq 3) was significantly less for those estimated from “above the knee” than “below the knee” (Chi square with Yates correction = 8.57, p = 0.003). These slopes were visualised as the two widest plateaus within the plot of running average slope vs log(*r)*. The strategy of finding the scaling region above the knee and below the knee did not work well as a large proportion of the VEPs did not result in PI≤0.3 (Table 2). Further, the magnitude of *D*_*2*_ was evaluated for a subset of VEPs analysed using *τ* = 16, *k* = 12 and excluding those VEPs which resulted in missing data (i.e. PI>0.3 for one or both below or above knee *D*_*2*_ estimates). Mean (SD) estimates of *D*_*2*_ from below the knee, and above the knee, were 4.02 (0.83) and 2.70 (0.35), respectively and were significantly different (F_1, 12_ = 25.15, p<0.0001). These findings are consistent with a previous study [25] on electrocardiograms that found the *D*_*2*_ estimate below the knee is usually higher than the *D*_*2*_ estimate from above the knee, although the significance of both was uncertain.

**Table 2:** Comparison of strategies for finding the scaling region.

VEPs (participant initials, group, sampling frequency at which VEP was recorded)	Strategy: Slope estimated from both below and above the knee in the plot of log(C(*r*)) vs log(*r*)				
	Number of VEPs with knees	Number of VEPs where PI≤0.3 based on *D*_*2*_		Number of VEPs where PI>0.3 based on *D*_*2*_	
		Below knee	Above knee	Below knee	Above knee
KL (control), 3606 Hz	12 out of 12	9	8	3	4
SC (control), 3606 Hz	11 out of 15	9	6	2	5
EP (amblyope), 3606 Hz	3 out of 4	4	2	0	1
RI (amblyope), 3606 Hz	16 out of 16	15	7	1	9
SC (control), 3606 Hz	11 out of 15	9	6	2	5
**VEPs**	**Strategy: Modified middle third rule**				
DL (control), 3606 Hz	12	12		0	
KL (control), 3606 Hz	12	11		1	
RB (control), 3606 Hz	13	13		0	
SC (control), 3606 Hz	15	15		0	
TK (control), 3606 Hz	12	12		0	
EK (control), 3606 Hz	13	13		0	
LW (control), 3606 Hz	5	5		0	
AA (amblyope), 3606 Hz	11	11		0	
AA2 (amblyope), 3606 Hz	15	15		0	
EP (amblyope), 3606 Hz	13	13		0	
ET (amblyope), 3606 Hz	13	13		0	
GG (amblyope), 3606 Hz	13	12		1	
JW (amblyope), 3606 Hz	14	13		1	
LM (amblyope), 3606 Hz	12	12		0	
MT (amblyope), 3606 Hz	12	12		0	
MG (amblyope), 3606 Hz	13	13		0	
NB (amblyope), 3606 Hz	12	12		0	
NM (amblyope), 3606 Hz	17	13		4	
RC (amblyope), 3606 Hz	12	12		0	
RI (amblyope), 3606 Hz	16	15		1	
RF (amblyope), 3606 Hz	13	13		0	
SB (amblyope), 3606 Hz	13	12		1	
YD (amblyope), 3606 Hz	12	12		0	
ZL (amblyope), 3606 Hz	13	13		0	
S1 (amblyope), 5000 Hz	4	4		0	

PI = Plateau Index. Shaded cells indicate abnormal VEPs.

It was not possible to reliably estimate *D*_*2*_ from the largest value in the widest plateau region in the plot of running average slope vs log(*r*) because it was difficult to define the extent of the plateau, particularly its start and end points. Sometimes, more than one plateau in a plot had the same width. Due to these ambiguities, the analysis could not continue to a *D*_*2*_ estimate. When an additional criterion was added that the widest plateau fell within the middle third of the plot [21], this resulted in reliable and unambiguous estimates with PI<0.3 (Table 2). This method is a modification of the Middle Third Rule [21].

#### Comparability of *D*_*2*_ estimates by sampling frequency and embedding dimension

**Fig 3:**
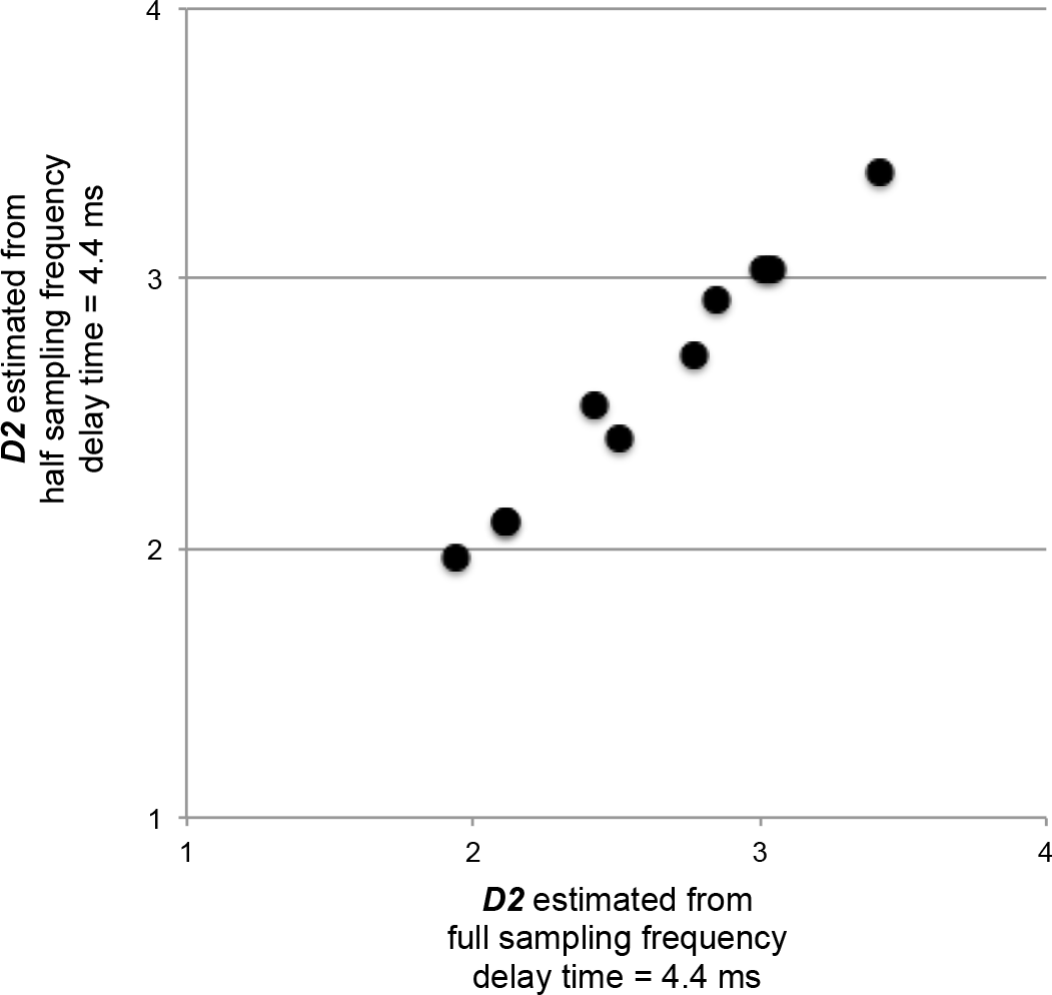
Scatterplot of *D*_*2*_ estimated from VEPs from full and halved sampling frequencies for the same delay times of 4.4 ms.

The correlation dimension was estimated using the optimised parameters and strategies (*τ* = 4.4 ms, 64 boxes and the optimised scaling method) for VEPs recorded at 5000 Hz and 3606 Hz at their original and halved sampling frequencies (2500 and 1803 Hz respectively) and compared. Estimates of *D*_*2*_ at original and halved sampling frequency were highly correlated R = 0.99 (Fig 3). There were no significant differences in the estimates of *D*_*2*_ (F_1, 9_ = 0.00, p = 0.99, mean difference = 0.00). These results were found by estimating fractal dimension from *D*_*2*_ at m = 7 for VEPs sampled at 3606 and 5000 Hz, *m* = 6 for 1803 Hz and *m* = 5 for 1000 Hz.

### Phase 2: Piloting the optimised method

The VEPs analysed were from a group of adults with normal (n = 4) and abnormal (n = 8) visual systems, recorded using the Medelec Synergy system with a 1000 Hz sampling frequency, single band pass filtered (1–50 Hz), with the 50 Hz notch filter applied to exclude mains electricity noise. The abnormal visual systems in the adults were due to a variety of causes including amblyopia, strabismus, pathological myopia and retinal vein occlusion. Visual evoked potentials in response to black-white (100% luminance contrast) and isoluminant magenta-cyan (42% chromatic contrast) gratings were analysed and compared between the normal and abnormal visual systems. Grassberger and Procaccia’s algorithm was implemented using *τ* as the closest approximation for 4.4 ms. The Shapiro-Wilk test [26] indicated the data were normally distributed. Analysis of variance was run with stimulus type (black-white or red-green) and viewing condition (monocular or binocular) as within subjects factors, group (normal or abnormal) as the between group factor and adjustment for multiple comparisons (Bonferroni correction). Estimated marginal means for *D*_*2*_ were 2.79 (95%CI 2.62–2.95) for the control group and 3.08 (95% CI 2.91–3.25) for the abnormal group and were found to be statistically significantly different (p = 0.02).

**Fig 4:**
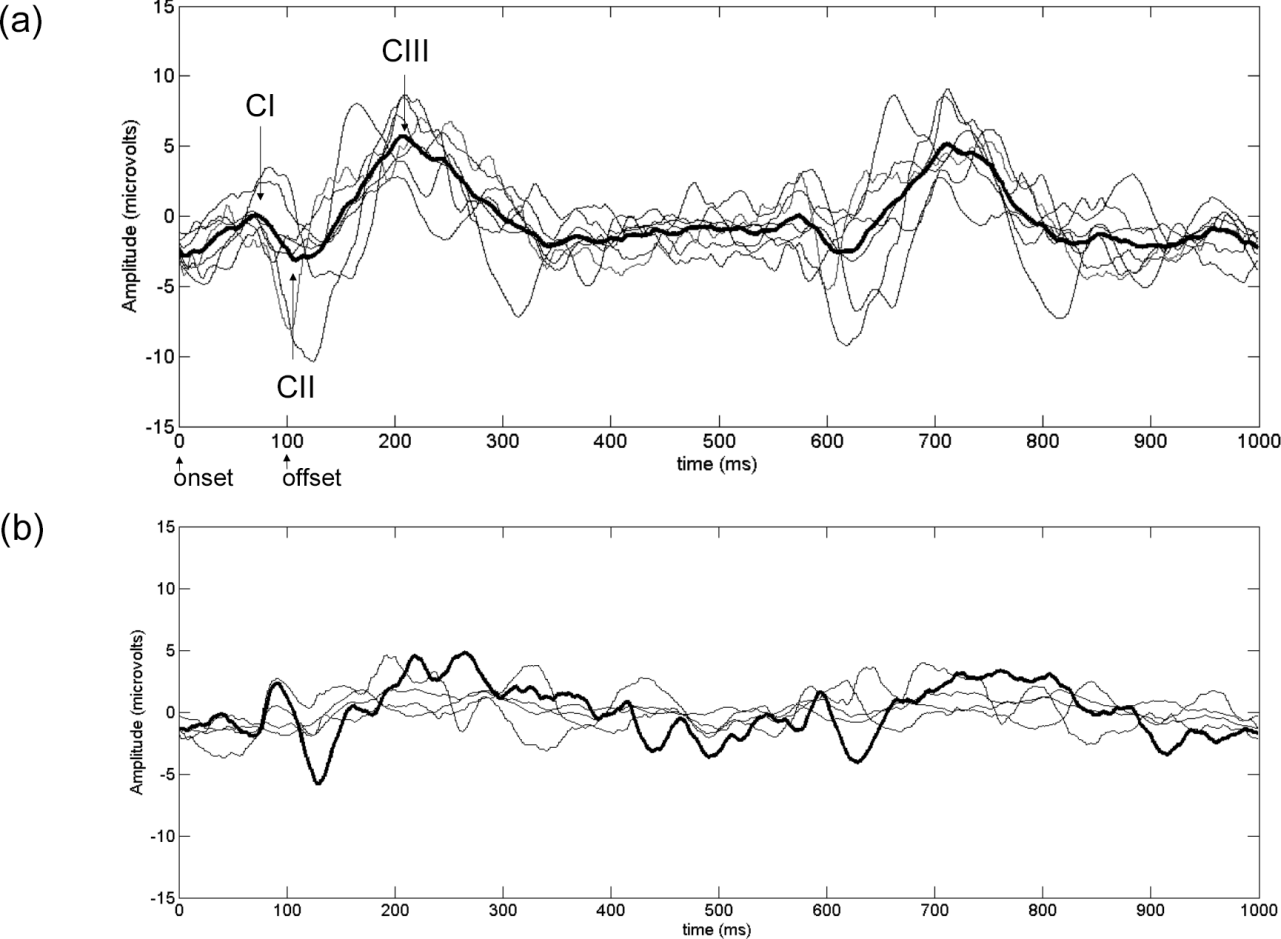
Adult binocular VEP responses to black and white pattern onset-offset grating stimuli. (a) Abnormal and (b) Normal adult binocular VEP responses. The components CI, CII and CIII and stimulus onset and offset times are shown for illustration purposes in (a). The group averaged responses are in bold. Individual responses, which were the average of two recordings, are presented as the thinner lines.

For descriptive purposes, Fig 4 presents the group average binocular VEPs in response to black-white 2 cpd sinusoidal gratings for the two adult groups. The standard ISCEV component CI, CII and CIII amplitudes and latencies were determined. Mean (SD) of VEP components are presented in Table 3.

**Table 3:** Group mean (SD) of VEP component amplitude and latency data.

Group	Stimulus	CI amplitude (μV)	CI latency (ms)	CII amplitude (μV)	CII latency (ms)	CIII amplitude (μV)	CIII latency (ms)
Adult Control	Black-white gratings	0.28 (1.77)	87.75 (14.52)	-1.42 (1.08)	117.00 (12.08)	2.67 (1.14)	193.50 (21.56)
Adult (Abnormal visual system)	Black-white gratings	1.38 (1.93)	76.00 (6.16)	-6.10 (3.14)	123.00 (10.07)	11.56 (5.82)	193.50 (19.76)
Child Control	Magenta-cyan gratings	1.77 (5.88)	63.52 (14.88)	-10.11 (8.62)	88.44 (25.74)	35.5 (8.23)	147.4 (6.9)
Child (Abnormal visual system)	Magenta-cyan gratings						175.38 (20.1)

The cells in grey indicate abnormal visual system data. Where components were not repeatable in latency for a group, mean (SD) are not reported for that group and the cells are shaded dark grey.

**Fig 5:**
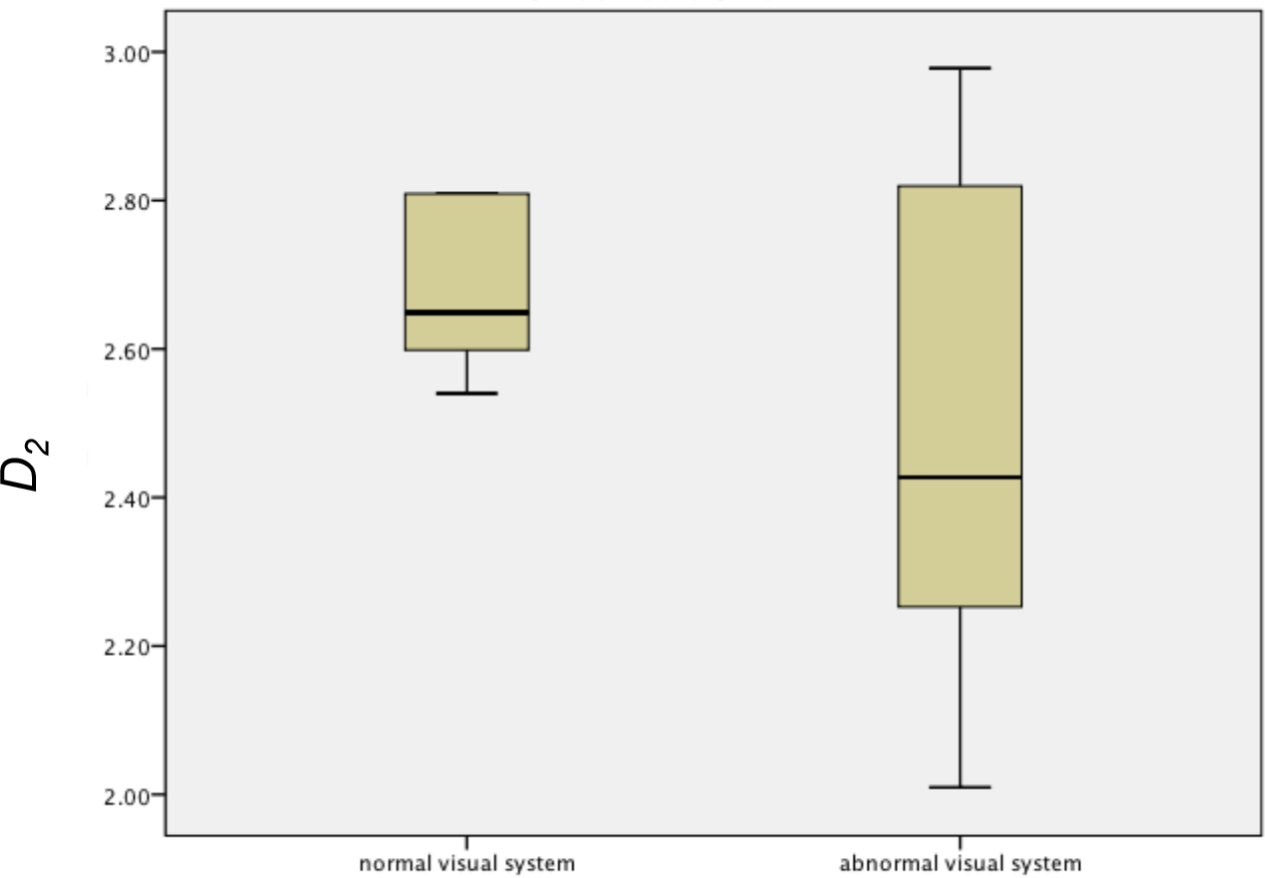
*D*_*2*_ estimated using a delay time of 4.4 ms of VEPs in response to binocular magenta-cyan stimulation in children with normal and abnormal visual systems.

**Fig 6:**
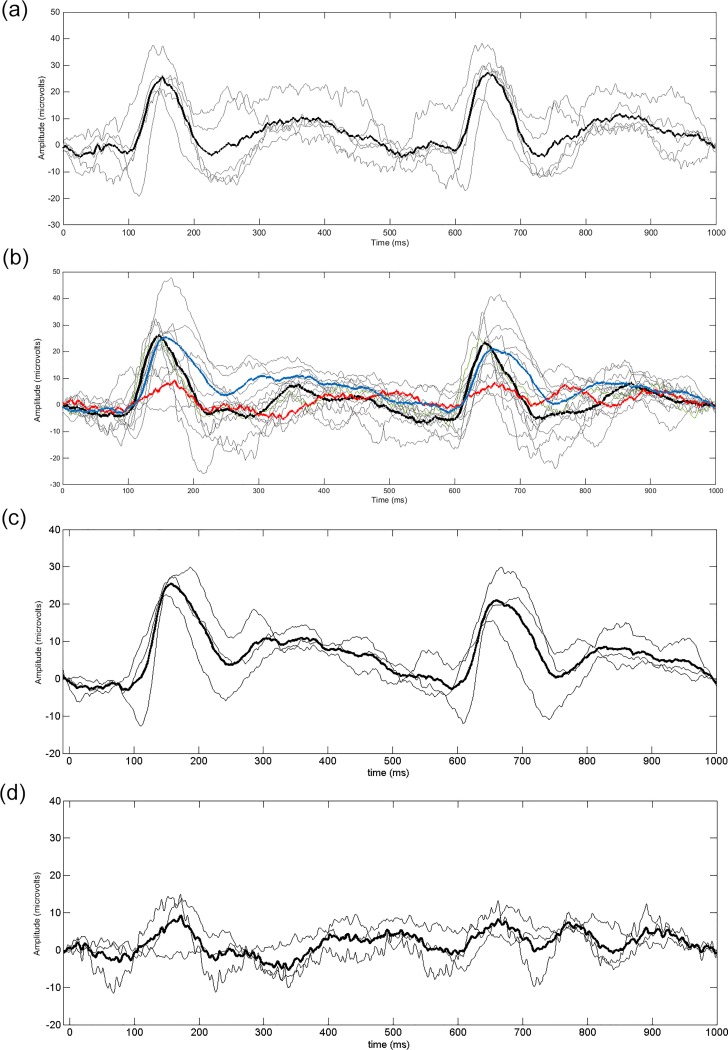
Children’s VEPs in response to magenta-cyan pattern-onset offset grating stimuli analysed using the optimised protocol. (a) shows the children’s VEPs drawn from children with normal visual systems. (b) shows the abnormal VEPs, with the average of the three lowest *D*_*2*_ VEPs in bold blue, the average of the three highest *D*_*2*_ VEPs in bold red, and the remainder of VEPs in bold black. Individual VEPs, which were the average of two recordings, are presented in grey. To illustrate the morphology of abnormal VEPs which tended towards the extremes of low and high *D*_*2*_ more clearly, the three abnormal VEPs with the lowest and highest *D*_*2*_ are shown in (c) and (d) respectively. The group averaged responses are in bold. Individual responses, which were the average of two recordings, are presented as the thinner lines.

Data comprising binocular VEPs in response to magenta-cyan isoluminant gratings recorded from a group of children with normal (n = 5) and abnormal visual systems (n = 13), with a 3606 Hz sampling frequency using a Diagnosys electrophysiology system, and no band pass filter or notch filter to exclude mains noise, were analysed. Grassberger and Procaccia’s algorithm was implemented using *τ* = 16, which corresponded to a delay time of 4.4 ms. All the children with abnormal visual systems had amblyopia associated with either strabismus or refractive error and were undergoing treatment. Although the Shapiro-Wilk test [26] indicated the data were normally distributed, Levene’s test [27] indicated that the variances were significantly different (Levene statstic_1,15_ = 7.76, p = 0.01), as illustrated in the boxplots in Fig 5. Hence the non-parametric independent samples median test was used to investigate between group differences, for which no significant difference in the medians was found (p = 1.0). Fig 5 shows that the abnormal group had more extreme tails than the normal group for *D*_*2*_. Fig 6 shows the children’s VEPs, including those that had the extremely high or low *D*_*2*_. CI and CII were not consistently repeatable. Therefore only the CIII component latencies could be reliably estimated for all children, as CIII amplitude depended on the CII component being repeatable (Table 3).

## Discussion

A central part of this study was to determine if useful protocols could be established for measuring *D*_*2*_ from VEPs of different sampling frequencies. The results confirmed a useful protocol could be established for all VEPs recorded at 1000, 3606 and 5000 Hz sampling frequencies. The results suggest that useful estimates of *D*_*2*_ may be obtained using the protocol described in this study.

The significance of the presence or absence of a “knee” remains unclear as knees were not always present and it was not always possible to obtain a reliable estimate of *D*_*2*_ using the slope above and below the knee. This suggests that trying to estimate *D*_*2*_ with reference to a knee is an unreliable method of estimating *D*_*2*_. The presence of two scaling regions separated by a knee, if they occurred, might reflect different physiological processes. In cases where they could be reliably measured, the mean difference between the slopes of the scaling regions below and above the knee was relatively large at 1.32, given that 0.5 is the difference between mature and immature visual systems, [6]. The scaling below the knee was not equivalent to *m* however it approached *m*, which may be consistent with an interpretation of data-limited space filling noise, for most of the VEPs. The scaling below the knee was more likely to demonstrate PI<0.3 (see Eq 3) than above the knee (see Table 2). The above suggests that trying to estimate *D*_*2*_ with reference to a knee is an unreliable method of estimating *D*_*2*_. Other possible explanations for the presence of a “knee” are either excessively high levels of correlation among nearby data points in the time series or use of an excessively large *τ* such that there is loss of correlation between components of points in phase space [24]. Excessively high or low levels of correlation for a given *τ* might indicate an unusual physiology. However, as VEPs from both amblyopic and normal visual systems were found to have knees while using a delay time of 4.4 ms, which appears to be appropriate using other criteria, it does not appear to reflect abnormality of physiology. Slope estimation from the widest plateau in the middle third is an alternative method which does not depend on the presence of a “knee”.

Within the limitations outlined, VEPs recorded from different electrophysiology systems using different sampling frequencies may yield comparable estimates of *D*_*2*_ provided the data are analysed within the parameters as outlined in this paper. However it must be noted that comparison of VEPs recorded from multiple set-ups or sites still requires care as it is likely that differences between laboratories extend to more than just the selection of sampling frequency. For example, differences in the kinds of equipment used such as electrode type, levels of electrical noise in the immediate surrounds and characteristics of the stimulus display may also impact on the morphology of the VEP recorded. In recognition of this fact, ISCEV acknowledges that VEP waveforms are expected to be similar across laboratories when recorded under standard conditions but when seeking to differentiate between normal and abnormal, each laboratory should establish its own set of normative values [1].

The protocols developed here appear to yield estimates of *D*_*2*_ for visual processing of stimuli that are comparable to estimates found in previous research [6]; ranging between 2 and 3.25 for binocular VEPs in response to magenta-cyan stimuli in children and adults. Taking the number of independent components composing a signal as the nearest integer above *D*_*2*_, this suggests that there may be somewhere between two and four independent components in these VEPs. This result also agrees with other analyses which measure the number of underlying components using different methods. For example, Maier et al. [12] used principal components analysis (PCA) on recordings between multiple scalp electrode sites to estimate the number of dipole sources that are active in the visual electrophysiological response to visual stimulation. They found that only two components are necessary to explain 95% of the power of the responses of the 24 electrodes they used for any stimulus type, and attributed the remaining 5% to noise. Almurshedi and Ismail [28] also determined that although PCA may yield five principal components (PCs), only the first two appeared to be strongly related to the signal. Zhang and Hood [29] found three significant principal components. They determined that, of these, the first (PC1) was likely to be a component derived purely from V1 of the brain, and that PC2 may be attributed to a small area of the visual cortex or derived from the extrastriate cortex, or a combination of the striate and extrastriate cortex.

Two kinds of stimuli were used which evaluated different aspects of the visual system. The black and white stimuli preferentially stimulated the magnocellular pathways whereas the magenta-cyan stimuli preferentially stimulated the parvocellular pathways of the visual system. In our sample of adults’ VEPs, *D*_*2*_ was found to be higher overall in the abnormal, than the normal visual systems. In contrast, the children with abnormal visual systems had VEPs in response to magenta-cyan stimuli which tended towards both higher and lower values of *D*_*2*_ than normal, but did not differ on average. For behavioural measures, values which tend towards extremes may be indicative of abnormal populations even though average responses are similar to normal populations e.g. to reflect the overactivity or underactivity or a system [30, 31].

Divergence between the adult and child findings might also be due to differences in the depth, type and severity of the visual system abnormality between the adults and the children. Adult participants exhibited long standing visual pathologies which ranged from ocular (e.g. vein occlusion) to cortical (e.g. amblyopia) and were resistant to different treatments. In contrast, the children in our study had only one type of abnormality (amblyopia) and were already undergoing treatment so the severity and depth of the abnormality was related to their compliance and response to ongoing treatment.

There is evidence that adaptations within the visual system in response to altered sensory inputs may take considerable time to reach a measureable effect. For example, Codina et al. [32] found cross-modal plasticity in individuals with longstanding deafness; they had high visual function associated with the recruitment of parts of the auditory cortex for visual processing. As dynamical variables may relate to the number of neuronal populations, the recruitment of additional neuronal populations towards processing would be evident as increased *D*_*2*_ [10, 11]. Interestingly, Codina et al. [32] found that cross-modal plasticity was only observed in adolescents from 13 years of age and adults, but not in children. Our data are consistent with Codina et al.’s [32] findings as group average differences in *D*_*2*_ are evident in the adult, but not the child, data.

The magnitude of *D*_*2*_ may be reflected in ISCEV morphology, however our results can only be suggestive rather than conclusive due to the small numbers in the high and low *D*_*2*_ groups in both the adult and child samples. Viewing Fig 6 and Table 3, it appears that for the children with abnormal systems and higher *D*_*2*_, CIII amplitude appeared low compared to the children with normal visual systems, and CI and CII were not consistently repeatable. In the children with abnormal visual systems and lower *D*_*2*_, the main difference from normal seemed to be the lack of a repeatable CI and CII. The CII component of the magenta-cyan VEP is typically less prominent in the immature visual system [33–35] hence their lack of repeatability in some of the abnormal VEPs might indicate changes in the development of the parvocellular pathways in those children. Studies suggest that CII may be more strongly related to the colour processing of the parvocellular system, whereas CIII is more related to the non-colour processing functions [34, 36]. The lack of repeatability in CI and CII components may indicate abnormalities in these aspects of cortical processing. The relationship between *D*_*2*_ and the magnitude of ISCEV VEP components requires further investigation.

This study has some limitations. As was discussed earlier, the ISCEV protocol for recording VEPs requires averaging to remove noise. Therefore some dynamics of brain processing are excluded by design. Overall brain function may impact on cortical processing of the visual signal, for example through attentional or feedback processes which may be reflected in EEG microstates [37]. Future research could look further into those aspects, however the present study has made a start by evaluating the averaged VEP.

While normal and abnormal VEP data was included to pilot the optimised protocol, it was stated in the introduction that a potential application would be to characterise normal and abnormal VEPs and ideally, *D*_*2*_ might even serve as an objective indicator of abnormality. Although the optimised protocol yielded unambiguous measures of *D*_*2*_ that were comparable to previous measures of *D*_*2*_ of the VEP and consistent with PCA estimates of numbers of components involved, indicating that the optimised protocol functioned as desired, there was a lack of clearly significant average differences between normal and abnormal VEP *D*_*2*_ measures in the children’s sample. This may be for a number of reasons. As discussed earlier, they might have already been recovering function. Additionally, the visual stimuli used in the present study preferentially stimulated chromatic parvocellular visual pathways that may have been relatively unaffected by the condition for the sample. Perhaps the use of different stimuli that evaluate other types of cortical processing such as achromatic parvocelullar function[38], might yield larger differences in processing which might then be reflected by greater differences in *D*_*2*_. As Sloper[39] noted, although amblyopia can be manifest as losses in parvocellular and magnocellular function, it is not a single uniform condition so the patterns of abnormalities will vary according to the types of abnormal visual experiences and the time periods in which they were experienced.

## Summary of Key Findings

**Fig 7:**
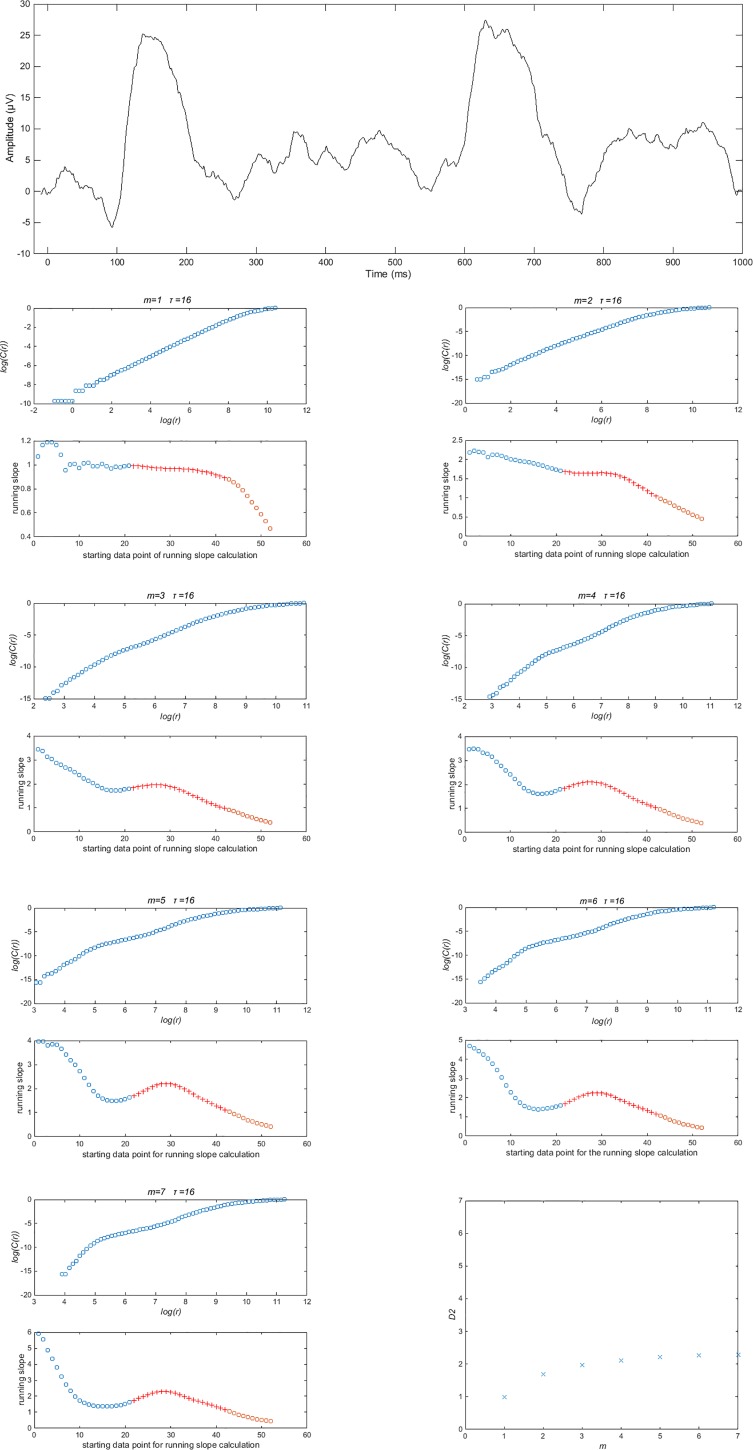
Depiction of the optimised analysis method. Top panel, example VEP. Intermediate panels show plots of log(*C*(*r*)) vs log(*r*) and plots of running average slope of log(*C*(*r*)) vs log(*r*) as a function of their ordered calculation for m = 1 to 7. The red crosses indicate the data derived from the middle third of the plot log(*C*(*r*)) vs log(*r*). The final panel is a plot of *D*_*2*_ for m = 1 to 7: the ceiling value at m = 7 is an indicator of the fractal dimension of the system.

To estimate the fractal dimension of transient visual evoked potentials for averaged recordings of one second duration and 2 Hz temporal frequency, the following optimised analysis protocol should be used when applying Grassberger and Procaccia’s algorithm:

1. Use a delay time, *τ*, which corresponds to 4 ms.
2. Use 64 bins of box sizes, *r*.
3. The embedding dimensions, *m*, for which *D*_*2*_ should be estimated for each VEP, includes 1, 2, 3…m* where m* is limited by the number of data points according to Eckman and Ruelle’s criterion (2log_10_(*N*)) [14]. Therefore, m* = 7 for 3606 and 5000 Hz sampling frequencies, m* = 6 for 1803 and 2500 Hz sampling frequencies and m* = 5 for 1000 Hz sampling frequency.
4. The scaling analysis strategy should be based on Henry et al.’s [21] and Tsonis’ [23] criteria and not consider the knee. For each *m*, running average slopes of 12 consecutive data points along the plot of log(*C*(*r*)) vs log(*r*) should be used to generate a plot of running average slope vs log(*r*) (Note log(*r)* is selected for its ordering value indicating the starting point on the plot of log(*C*(*r*)) vs log(*r*) from which the running average slope was calculated, rather than for its numerical value. In Fig 7, the running average slopes are plotted as functions of their ordered calculation). The widest plateau in that plot should be identified. If the widest plateau falls within the middle third of the plot (indicated as the red crosses in Fig 7), then the highest value for slope in the plateau within the middle third is the estimate of *D*_*2*_ for that *m*.
5. Next, plot *D*_*2*_ as a function of *m* (see bottom right panel in Fig 7). If *D*_*2*_ plateaus as it approaches m*, using the criterion that PI<0.3 [5], then *D*_*2*_ at m* may be used as an estimate of the fractal dimension of the underlying system which produced that VEP.
6. Piloting the optimised method indicated *D*_*2*_ has the potential to increase understanding of visual processing in normal and abnormal visual systems.

## Conclusions

In conclusion, VEPs recorded from different electrophysiological recording systems using different sampling frequencies may yield comparable estimates of *D*_*2*_ provided the data is analysed within the protocols outlined in this paper. This will assist in the comparison of *D*_*2*_ measures between electrophysiology systems which employ different sampling frequencies, which enhances its applicability as a complementary objective measure of visual system electrophysiological activity as imaged using the VEP. Interpreting the nearest integer above *D*_*2*_ as the minimum number of independent components comprising the signal, is broadly consistent with the results of principal component analysis. The development of a robust protocol for measuring *D*_*2*,_ as undertaken here is an important step towards using *D*_*2*_ measurements clinically.
